# Advancing Metal–Organic Framework‐Based Composites for Effective Chemical Warfare Agent Detoxification under Real‐World Conditions

**DOI:** 10.1002/adma.202413848

**Published:** 2025-04-10

**Authors:** Zhihua Cheng, Kira M. Fahy, Gregory W. Peterson, Kent O. Kirlikovali, Omar K. Farha

**Affiliations:** ^1^ Department of Chemistry Northwestern University 633 Clark Street Evanston IL 60208 USA; ^2^ International Institute for Nanotechnology (IIN) Northwestern University 633 Clark Street Evanston IL 60208 USA; ^3^ Paula M. Trienens Institute for Sustainability and Energy Northwestern University Evanston IL 60208 USA; ^4^ Department of Chemical and Biological Engineering Northwestern University 633 Clark Street Evanston IL 60208 USA; ^5^ U.S. Army Combat Capabilities Development Command Chemical Biological Center 8198 Blackhawk Road Aberdeen Proving Ground MD 21010 USA

**Keywords:** chemical warfare agents detoxification, designing principle, metal–organic frameworks/textile composite, real‐world condition

## Abstract

Threats from toxic chemical warfare agents (CWAs) persist due to war and terrorist attacks, endangering both human beings and the environment. Metal–organic frameworks (MOFs), which feature ordered pore structures and excellent tunability at both metal/metal cluster nodes and organic linkers, are regarded as the best candidates to directly remove CWAs and their simulants via both physical adsorption and chemically catalyzed hydrolysis or oxidization. MOFs have attracted significant attention in the last two decades that has resulted from the rapid development of MOF‐based materials in both fundamental research and real‐world applications. In this review, the authors focus on the recent advancements in designing and constructing functional MOF‐based materials toward CWAs detoxification and discuss how to bridge the gap between fundamental science and real‐world applications. With detailed summaries from different points of view, this review provides insights into design rules for developing next‐generation MOF‐based materials for protection from both organophosphorus and organosulfur CWAs to mitigate potential threats from CWAs used in wars and terrorism attacks.

## Graphical abstract

This review describes recent developments in the design and synthesis of metal–organic frameworks (MOF)/textile composites for the detoxification of chemical warfare agent and simulants with extensive discussion on the advantages and disadvantages of different methods. It also summarizes design rules for more active MOF catalysts and provides the implementation principles to achieve desired performances in practical conditions.

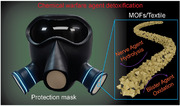

## 1 Introduction

The threats of exposure to toxic chemical warfare agents (CWAs) have increased in recent years despite efforts by the Chemical Weapons Convention to prohibit their use, especially due to the increased global war crisis and terrorist attacks.^[^ ^1^ ^]^ Currently, risk of CWA exposure arises from two sources: remaining stockpiles from past and use by terrorist organizations, with additional risks posed by the discovery of buried or undeclared chemical weapons.^[^ ^1^, ^2^ ^]^ Both have posed a huge threat to civilians, animals, and the environment on a global scale. Therefore, the design and fabrication of functional materials that are capable of safely and efficiently capturing and degrading CWAs is critical to alleviate the potential threats to humankind and the environment.^[^ ^1^, ^2^, ^3^ ^]^ In this regard, porous materials like activated carbon, zeolites, metal oxides, and metal–organic frameworks (MOFs) have emerged as suitable protective materials due to their high surface areas and unique surface properties.^[^ ^1^ ^]^ For example, activated carbon has been widely used as a cheap adsorbent for protection against CWAs since world war I. However, weak physical interactions between carbon surfaces and CWAs, as well as the ill‐defined pore structures in activated carbon, result in very low CWA uptake capacities, rendering these materials unable to efficiently address threats. In addition, even with state‐of‐the‐art carbon‐based materials, their inability to break down toxic chemicals renders them temporary storage solutions that face the risk of leaching, which can potentially contaminate personnel or communities if treated or transported improperly. Therefore, it is important for protective materials to be able to both capture and catalytically deactivate these toxic chemicals to eliminate their threat. MOFs, which are atomically precise organic–inorganic hybrid structures that form from the coordination of metal ion or cluster secondary building units (SBUs) with organic linkers (or ligands), comprise a class of hybrid materials that are capable of meeting this challenge. For instance, they offer an advantageous platform for directly capturing and degrading toxic chemicals and gases within their ordered and tunable micro‐ and meso‐porous cages and channels.^[^ ^1^, ^3^, ^4^ ^]^ Due to their tunable properties through modification of both the metal nodes and organic linkers, MOF‐based materials have immense structural diversity and a plethora of applications in chemistry, chemical engineering, biology, and even mechanics. Most notably is their potential for gas capture, separation, storage, catalysis, biomedicine, and sensing.

In practical applications, MOFs offer significant opportunities for incorporation into protective systems, including filters, protective suits, and decontamination operations. Current filters made from activated carbon typically adsorb CWAs but do not detoxify them, which can lead to off‐gassing hazards over time. Similarly, protective suits often utilize activated carbon beads, which also lack significant reactivity toward CWAs, posing risks of off‐gassing or direct contact hazards post‐exposure. In contrast, MOFs provide the potential for both high adsorption and catalytic detoxification, making them highly advantageous for these applications. MOFs are uniquely suited for these applications due to their unparalleled tunability, allowing precise control over pore size, adsorption capacity, and catalytic activity, depending on the specific detoxification needs. Perhaps the most promising transition for MOFs is in decontamination operations. These range from powder applications for skin decontamination, similar to reactive skin decontamination lotion (RSDL), to wipes for removing agents from surfaces and equipment, such as the joint service equipment wipe (JSEW). MOFs could also be used in sprayable slurries for decontaminating vehicles and large surfaces or for the elimination of large chemical stockpiles. Decontamination operations often require large amounts of water, which may not be readily available, so developing MOF powders or composites that can effectively detoxify agents without water presents a significant advantage.

The tunable properties of MOFs enable optimization for specific use cases. For instance, in protective suits, the priority is high adsorption capacity, as adsorption is the primary mechanism for removing CWAs. Incorporating active catalytic sites into the material could enhance reactivity, removing agents as a contact hazard and potentially allowing the suit to be regenerated or reused after exposure. In protective suits, for instance, active catalytic sites could potentially enable in situ regeneration, allowing the material to detoxify adsorbed agents and restore its protective function. While protective suits prioritize adsorption, other use cases, such as the decontamination of stockpile agents, may benefit more from catalytic activity. Here, small amounts of MOF or MOF‐based composites could be introduced to catalyze detoxification over time, with external surface reactivity being more critical than adsorption capacity, which could inhibit pore accessibility and reduce reactivity. These use cases illustrate the versatility of MOFs, which can be designed for both adsorption and catalysis depending on the application. Beyond the chemistry advantages, MOFs can be anchored to textiles through direct growth or post‐synthetic grafting to allow for seamless integration into protective fabrics, enhancing their functionality without sacrificing flexibility or comfort.

**Figure 1:**
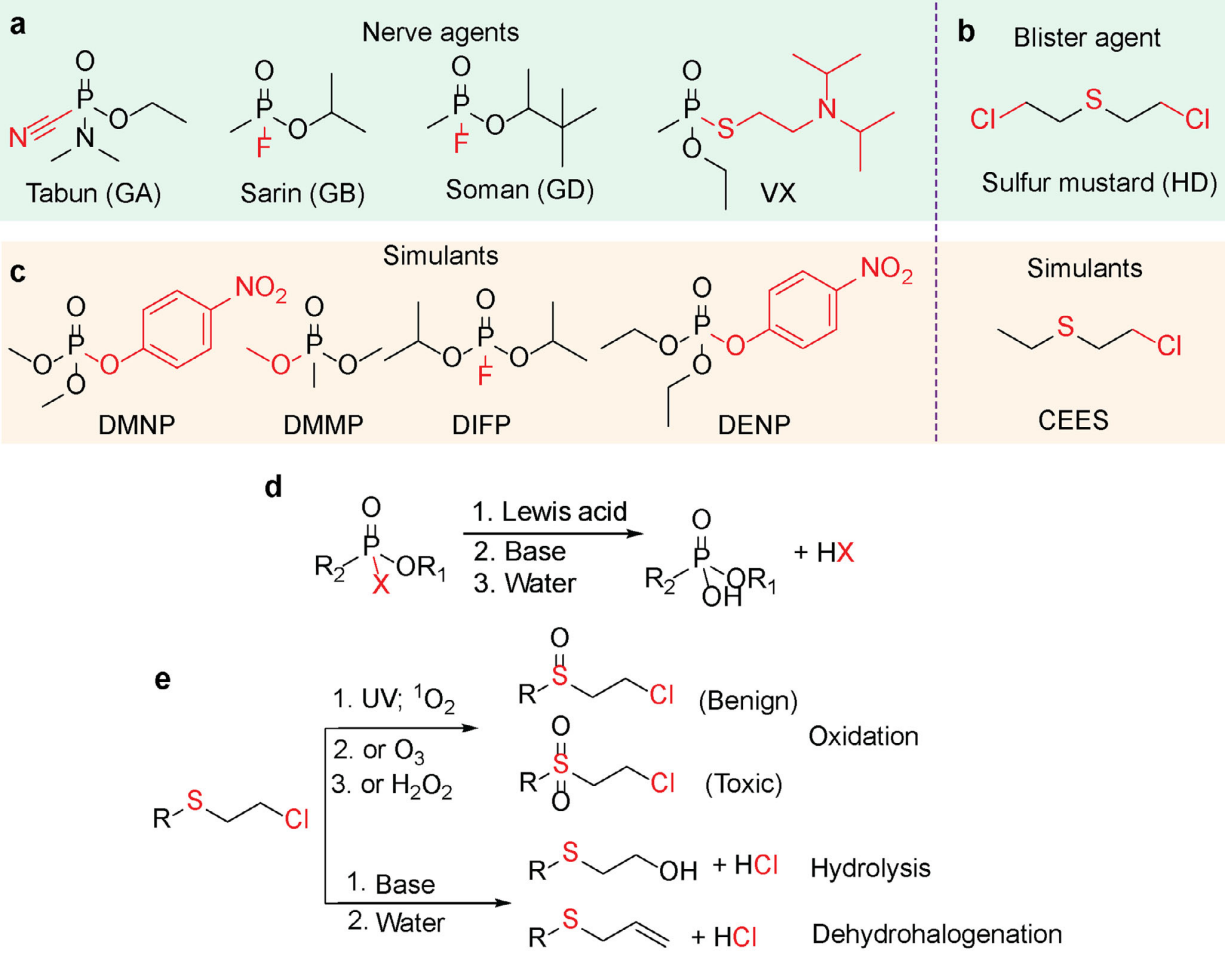
Chemical structures of CWAs and their simulants. a) Typical nerve agents; and b) blistering agents in CWA weapons; c) the corresponding CWA simulants discussed throughout this article. The possible detoxification sites have been marked in red; d) the hydrolysis of nerve agent and simulants through Lewis acidic sites in the presence of base and water; e) three possible reaction pathways to detoxify blistering agents and simulants by oxidation, hydrolysis, and dehydrohalogenation.

In order to fully understand how MOFs can be optimized for CWA detoxification, it is important to consider the chemical structures and reaction pathways involved in the detoxification of these agents. Similar to other chemical processes happening inside of pores of MOFs, detoxification of CWAs necessitates adequate pore size and active sites that allow either physical or chemical adsorption and desorption. Therefore, the structure of CWA molecules and their bonding moieties must be considered. While there are many categories of CWAs, the two most toxic and widely recognized classes are organophosphorus nerve agents and organosulfur blistering agents, as shown in **Figure** **1**. The former is mainly composed of phosphonate‐based chemicals, including the G‐series agents tabun (GA), sarin (GB), and soman (GD), as well as the V‐series venomous agent X (VX), as typical examples (Figure 1a). These compounds are considered to be the most lethal synthetic nerve agents due to their mechanism in which they irreversibly inhibit the acetylcholinesterase enzyme, which prohibits the breakdown of the neurotransmitter acetylcholine and results in asphyxiation within minutes.^[^ ^1^, ^4^, ^5^ ^]^ In contrast, blistering agents or vesicant agents, such as mustard (sulfur mustard, HD, Figure 1b), result in severe chemical burns upon exposure to the skin, eyes, lungs, or any other mucous membrane.^[^ ^4^, ^6^ ^]^ Due to the high risk from these CWAs, a limited number of research labs around the world have the credentials to perform detoxification experiments with the real CWAs, which decreases the output needed to develop cost‐effective catalysts. As a result, researchers employ CWA simulants (Figure 1c), which mimic the structures of actual agents but lack their acute toxicity, as accepted replacements for CWAs in fundamental studies.

Nerve agents and blistering agents feature diverse chemical structures and necessitate different approaches in the context of designing active catalytic MOF‐based materials.^[^ ^4^, ^6^, ^7^ ^]^ For instance, hydrolysis of organophosphorus nerve agents and their simulants proceeds highly efficiently and affords benign products (Figure 1d).^[^ ^1^, ^8^ ^]^ In contrast, organosulfur blistering agents and their simulants can be detoxified through the selective oxidation of the sulfur to form a sulfoxide, hydrolysis of the C─Cl bond to form a terminal alcohol, or dehydrohalogenation of the C─Cl bond to form a terminal alkene (Figure 1e).^[^ ^6^, ^9^ ^]^ Formation of the sulfoxide requires either the use of a chemical oxidant (e.g., H_2_O_2_,^[^ ^10^ ^]^ O_3_^[^ ^1^, ^11^ ^]^) or singlet oxygen (^1^O_2_),^[^ ^9^, ^12^ ^]^ which can be produced upon absorption of ultraviolet or visible light by a functional moiety on the linker of the MOF (e.g., pyrene^[^ ^1^, ^9^, ^10^, ^12^ ^]^ and porphyrin^[^ ^9^, ^10^, ^13^ ^]^). Notably, singlet oxygen has been shown to selectively form the desired sulfoxide produce and does not form the doubly oxidized sulfone product, which is undesired because it exhibits a similar toxicity to that of the initial organosulfur CWA or simulant (Figure 1e). Conversely, the substitution and dehydrohalogenation pathways involve the participation of water and base, similar to the detoxification of organophosphorus nerve agents. Overall, the existence of multiple viable CWA detoxification presents an opportunity for the development of tailored strategies for each class of CWAs. This review summarizes recent advances and provides a systematic discussion of design principles and strategies to guide the future development of efficient MOF‐based catalysts for practical CWA detoxification.

### 1.1 Designing Rules for Active MOF‐Based Catalysts toward CWA Detoxification

The development of MOF‐based CWA detoxification catalysts can be divided into two stages. In the first stage, researchers devoted their efforts to discovering, designing, and engineering the structures of MOFs to improve their catalytic performance. This includes investigating different metal centers, particularly metal clusters or SBUs,^[^ ^14^ ^]^ and finely tuning the organic linkers to mimic the active sites of the natural enzyme phosphotriesterase, which resulted in increased performance toward nerve agent detoxification.^[^ ^1^, ^4^, ^14^, ^15^ ^]^ Among the different metal centers explored by researchers, zirconium‐based MOFs (Zr‐MOFs) showed the most promising performance for the hydrolysis of nerve agents and their simulants and will comprise a main focus throughout this review.^[^ ^3^, ^16^ ^]^ Based on extensive research in this area, researchers have proposed underlying hydrolysis mechanisms and possible pathways to improve catalytic activity. This fundamental understanding has been well‐established in our previously reported reviews, which summarize key design rules that guide the current and future development in this field.^[^ ^1^, ^2^, ^3^, ^4^, ^6^, ^17^ ^]^ Despite this extensive understanding, however, these works have predominately focused on understanding detoxification reactions for CWAs and their simulants in solution, which is relevant for the removal of chemical weapon stockpiles but cannot guarantee comparable performance in the solid phase that is required to implement these materials in the field. In fact, the catalytic performance of MOF‐based materials at gas‐solid interfaces decreases significantly in comparison to their performance in solution. This gap needs to be bridged for the future development of protective suits and devices that can be used by civilians and personnel exposed to these chemical threats.

The second stage of the development of MOF‐based CWA detoxification catalysts builds upon these initial results and explores the synthesis of MOF‐based composites, which have been developed using fabrics, textiles, breathable rubber films or barriers, or beads, that can decompose toxic chemicals under practical conditions.^[^ ^3^, ^4^, ^18^ ^]^ For example, after early studies demonstrated that Zr‐MOFs were capable of rapidly hydrolyzing nerve agent simulants in basic aqueous solutions, researchers sought to translate this performance to fully solid‐state systems in which MOF/fiber composites that contain heterogeneous bases could detoxify nerve agent simulants using water that is present in the air as humidity. However, closing this gap between traditional fundamental studies and practical applications begets new parameters that need addressing. Therefore, in this work, we will discuss the recent progress that bridges fundamental research and practical applications in detoxification of CWAs and simulants.

To effectively bridge the gap between fundamental research and real‐world applications, a range of evaluation techniques is necessary to assess MOFs and their corresponding composites at different stages of development. These techniques vary in complexity, progressing from fundamental studies to more practical testing in real‐world conditions. At the most fundamental level, surface science experiments, such as infrared spectroscopy under ultra‐high vacuum,^[^ ^19^ ^]^ can probe interactions between CWAs and MOF active sites, revealing key interactions and energetics to provide insight into how adsorption and desorption mechanisms can be optimized for detoxification. In initial performance evaluations, solution‐based stirred tank reactor tests are conducted in which solutions containing the simulants are added to suspensions of the MOF or composite. These parameters provide the most ideal conditions for catalysis as diffusion is essentially instantaneous, allowing for rapid assessment of catalytic reactivity, turnover, and reaction kinetics. Following these studies, analogous experiments can be performed using the real agents to better assess catalyst performance. For more realistic conditions, liquid or vapor dosing tests are employed in which the agent is applied to a solid MOF composite and allowed to react over time. After incubation, the material is extracted or digested to quantify residual agent and degradation products. Solid‐state nuclear magnetic resonance (NMR) spectroscopy can also be used to monitor the in situ breakdown of the agent and the formation of byproducts. Together, these methods offer a more accurate evaluation of the material's catalytic efficiency, reaction rates, and the total amount of agent removed under non‐ideal conditions. At the most advanced stages, practical testing methods such as chemical breakthrough,^[^ ^20^ ^]^ permeation,^[^ ^21^ ^]^ and decontamination^[^ ^22^ ^]^ tests simulate actual use cases in real‐world scenarios, including high humidity and continuous CWA exposure. These tests offer critical data for evaluating the effectiveness of MOF‐based materials in practical applications, such as protective gear.

**Figure 2:**
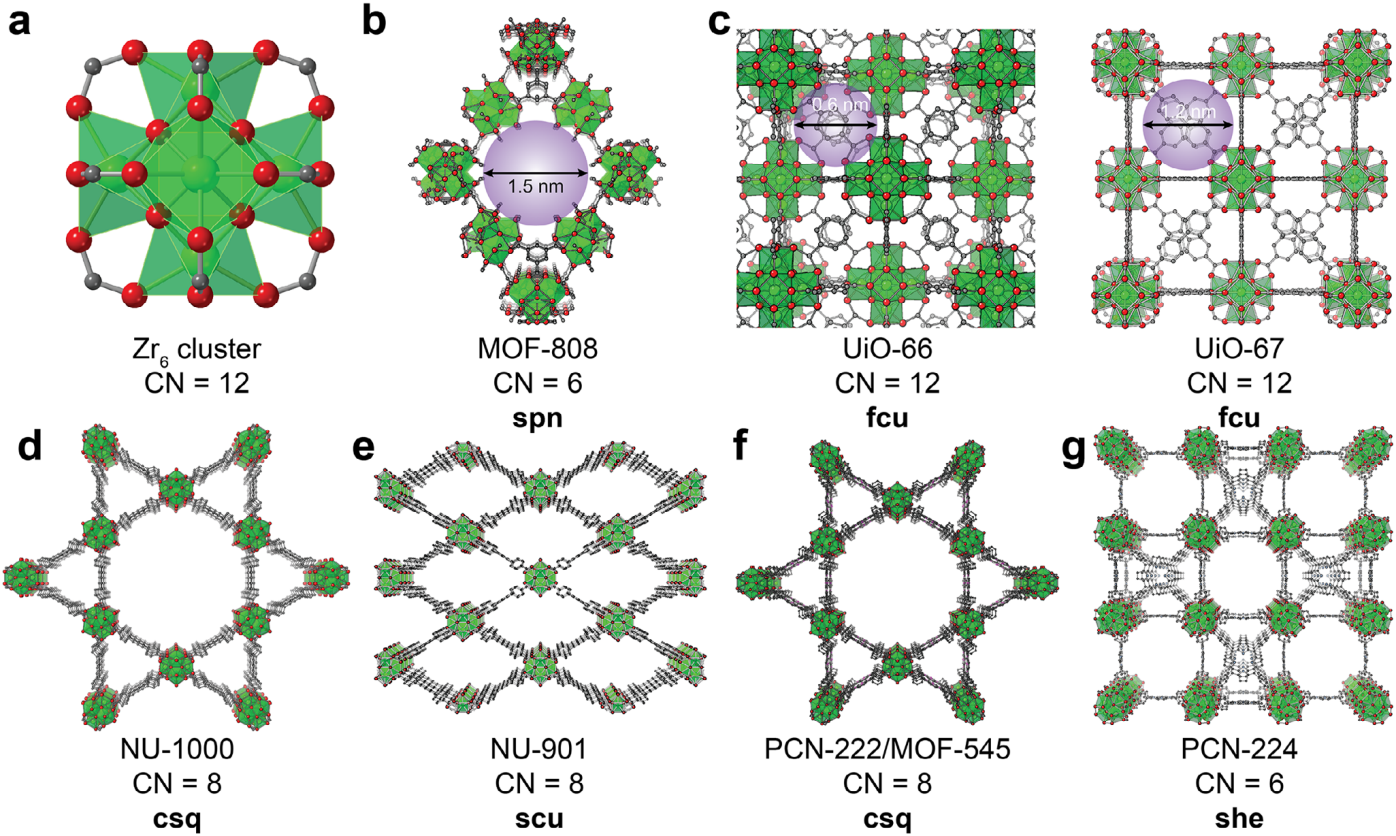
The typical Zr‐based MOFs constructed from Zr_6_ clusters with different topologies and coordination numbers (CNs). a) Zr_6_ clusters, Zr_6_(μ_3_‐O)_4_(μ_3_‐OH)_4_, with CN of 12. b) MOF‐808 with CN of 6, **spn** net, and pore size of 1.5 nm; c) UiO‐66 and its isoreticular UiO‐67 with CN of 12 and **fcu** topology, which is formed by modifying the length of linker; d–g) Other Zr‐based MOFs with different CNs (8 and 6) and different topologies (**csq** for NU‐1000 and PCN‐222 or MOF‐545; **scu** for NU‐901 and **she** for PCN‐224). Among all the MOFs, MOF‐808 is most widely used due to its excellent properties emerging from a relatively low CN and rigid framework. All hydrogen atoms are omitted intentionally for better visualization.

Before discussing the recent development of efficient materials toward removing CWA threats under practical conditions, we first provide underlying knowledge necessary for thoughtfully evaluating MOF‐based CWA detoxification catalysts, including performance evaluation and design principles of functional MOFs. As an example, MOF‐808 (**Figure** **2**) has been tested for its ability to hydrolyze the nerve agent simulant dimethyl‐4‐nitrophenyl phosphate (DMNP). Both solution‐based and solid‐phase dosing methods have demonstrated its catalytic potential, allowing us to elaborate on these key concepts. To evaluate the performance of MOF‐based materials in the detoxification of CWAs and their simulants, one key performance parameter is the half‐life (*t*_1/2_) of decomposition for each specific CWA or simulant. This is a measurement of the reaction time needed to remove half of the CWA or simulant molecules and is used as a metric for the catalytic efficiency of the MOF for these reactions. The smaller the *t*_1/2_, the higher the reactivity and degradation rate of the catalyst toward CWAs. In the context of detoxifying organophosphorus CWAs and their simulants, previous studies have illustrated that the *t*_1/2_ of hydrolysis using MOF‐based catalysts that feature Lewis acidic active sites at the metal nodes, such as Zr‐MOFs, can be significantly improved through the following design rules:^[^ ^1^, ^23^ ^]^ 1) increasing defects or lowering the coordination number of SBUs to increase the number of active sites per node;^[^ ^16^, ^24^ ^]^ 2) increasing the accessibility of active sites by constructing favorable pore sizes and apertures to optimize reaction kinetics and enhance mass transport;^[^ ^25^ ^]^ 3) decreasing the MOF particle size to increase the specific surface area;^[^ ^9^, ^24^, ^26^ ^]^ and 4) introducing non‐chemical interactions (e.g., hydrogen bonds) between CWAs and organic linkers to facilitate chemical reactions, as suggested by the catalytic performance of UiO‐66‐NH_2_ relative to that of the parent UiO‐66 that results from enhanced hydrogen bonding interactions between DMNP and the amino groups on the linkers of UiO‐66‐NH_2_.^[^ ^27^ ^]^

The performance enhancements enabled through these design rules can be evidenced in the use of nano‐sized MOF‐808 (≈200 nm particles) for DMNP hydrolysis, which exhibits a *t*_1/2_ of hydrolysis of <0.5 min in basic aqueous solutions, positioning this Zr‐MOF as the state‐of‐the‐art organophosphorus CWA detoxification catalyst under these conditions.^[^ ^28^ ^]^ This short *t*_1/2_ was achieved by synergistically tuning the key design rules summarized above. First, MOF‐808 is composed of Zr_6_‐based nodes (Zr_6_(μ_3_‐O)_4_(μ_3_‐OH)_4_, Figure 2a),^[^ ^1^, ^16^, ^29^ ^]^ which feature Lewis acidic Zr(IV) metal centers that are bridged by hydroxy and oxo groups and are reminiscent of the Zn─OH─Zn active site in the enzyme PTE.^[^ ^2^, ^4^, ^15^, ^17^, ^18^, ^30^ ^]^ Second, MOF‐808 features a relatively large pore (1.5 nm) and aperture size (1.85 nm) (Figure 2b) in comparison to those of UiO‐66 (0.6 nm pore size, 1.1 nm aperture size) with a similar linker length. The open pores and aperture windows facilitate the permeability and accessibility of DMNP molecules toward the node‐based active sites. In contrast, UiO‐66 (Figure 2c) exhibits relatively limited surface permeability, thus showing lower performance with *t*_1/2_ of ≈45 min toward the hydrolysis of DMNP under similar conditions. This design rule has been verified with the isoreticular UiO‐67,^[^ ^16^, ^31^ ^]^ which contains the longer biphenyl‐4,4′‐dicarboxylic acid linker and shows an improved *t*_1/2_ of <4.5 min (Figure 2c). Third, the **spn** topology of MOF‐808 affords Zr_6_ clusters with a coordination number (CN) of 6 between the Zr_6_ clusters and tritopic benzene‐1,3,5‐tricarboxylic linkers (i.e., 6‐connected nodes). Since the connectivity for an ideal Zr_6_‐based node is 12‐connected, this means an ideal node in MOF‐808 features 6 unsaturated Lewis acidic active sites that are available react with nerve agents and their simulants. This low CN is key to improving the intrinsic activity toward nerve agent detoxification and is supported by extensive studies on defect engineering of Zr‐MOFs and designing various MOF with different topologies (Figure 2b–g).^[^ ^1^, ^16^ ^]^ Fourth, the high stability of Zr‐MOFs under basic conditions facilitates catalysis since hydroxide ions are likely responsible for the nucleophilic attack of the coordinated organophosphorus compound, and hydroxide ions potentially help facilitate regeneration of the active sites to enable catalytic turnover.^[^ ^1^ ^]^ Therefore, reactions are typically conducted at alkaline pH values to ensure enough hydroxide ions are present in solution (usually at pH ≈10). In this respect, the high base stability of Zr‐based MOFs is advantageous. Finally, MOF‐808 has a relatively small particle size, engendering greater surface area and defects. This enhances the permeability of DMNP molecules within the pores, facilitating reaction kinetics and mass transport. Taken together, these characteristics are all important in positioning MOF‐808 as an advantageous catalyst for CWA detoxification.

Apart from *t*_1/2_, other parameters like conversion rate^[^ ^32^ ^]^ (the total conversion of reagents at specific time) and turnover frequency^[^ ^16^ ^]^ (TOF, the number of chemical reactions occurring per active site per second) are also commonly used. However, the former reflects the average rate of CWAs that have been converted into non‐toxic chemicals over a longer period, which has been widely used to determine the reactivity of MOFs toward degradation of blistering agents and their simulants. It is important to mention that this parameter cannot be used to indicate the efficiency of catalyst with a high temporal resolution in comparison to *t*_1/2_. On the contrary, the TOF also reflects the reaction rate and intrinsic catalytic activity; however, the number of active sites that participate in CWA detoxification is ambiguous in this field since it is often unclear whether all metal centers participate in the catalysis, including those in the interior of the crystallite, or just metal centers that are close to the crystallite surface. This inconsistency in different reports results in an inequitable comparison of the intrinsic activities, so *t*_1/2_ is widely used as a reference to indicate the reactivity of MOFs.

It is important to emphasize that the design rules summarized previously pertain to work in detoxification of nerve agents and their simulants. However, these design rules are also applicable to the hydrolysis and dehydrohalogenation of organosulfur blistering agents, such as bis(2‐chloroethyl)sulfide (HD), and their simulants, such as 2‐chloroethyl ethylsulfide (CEES), since these reactions proceed under similar conditions (i.e., in the presence of base and water). However, oxidation of organosulfur compounds requires different conditions, and the selective oxidation to sulfoxide species proceeds effectively through a radical pathway. One strategy to generate radicals is to directly incorporate functional groups that can produce the radical oxidation species^[^ ^33^ ^]^ (i.e., polyoxometalates [POMs] or persulfate moieties). This strategy requires a delicate balance of the oxidation strength of the reagent to achieve controllable conversion without over‐oxidation of the organosulfur compound to the highly toxic sulfone products (e.g., CEES to CEESO_2_).^[^ ^6^, ^9^, ^10^, ^34^ ^]^ Alternatively, an indirect method to generate reactive species involves the conversion of triplet oxygen (^3^O_2_) into singlet oxygen (^1^O_2_), which has been demonstrated to selectively generate the non‐toxic sulfoxide product CEESO. For this detoxification strategy, the formation of ^1^O_2_ involves an energy transfer by photoexcitation, so MOFs comprised of linkers with photosensitive functional groups (e.g., pyrene^[^ ^1^, ^9^, ^10^, ^12^ ^]^ and porphyrin^[^ ^9^, ^10^, ^13^ ^]^) are active under the required reaction conditions.

Significant efforts have been made to improve the catalytic activities of MOFs toward the detoxification of both organophosphorus and organosulfur CWAs, establishing well‐characterized design rules at the laboratory scale. While these helps inform the design for practical use, application‐based research is required to bring this technology to the field. Subsequent studies are investigating how to circumvent the need for large amounts of base, water, and oxidants to allow for detoxification in the field. Another important body of research explores the optimal methods for integration of these powder‐formed catalysts into functional composite materials and products. In order to produce composite materials with high activities in practical settings, this field must bridge the gap between fundamental and applied research. In the next sections, we will discuss the recent developments of how to link fundamental research and real‐world applications through hybridizing MOF‐based materials with textiles to achieve the detoxification of CWAs under practical conditions.

### 1.2 Fabrication of MOF/Textile Composites for CWA Detoxification

The development of powder MOF‐based detoxification materials for removing CWA stockpiles necessitates their viability in practical settings. Integrating active MOF‐based materials into protective gear, such as masks and suits, requires designing wearable fabrics and textiles with embedded MOFs.^[^ ^3^, ^4^, ^18^ ^]^ This approach directly protects personnel by creating a protective layer that prevents direct exposure to CWAs. Apart from the engineering challenges associated with constructing MOF‐based textile composites with enough mass loading to achieve sufficient protection with both high efficiency and stability, other properties like gas/water permeability,^[^ ^35^ ^]^ as well as flexibility and durability,^[^ ^26^, ^36^ ^]^ must also be considered. For example, due to the lack of sufficient water at these gas/solid interfaces, the ability for these materials to leverage humidity from the air must be improved to facilitate hydrolysis and active site regeneration.

Until now, various strategies have been developed for the preparation of MOF/textile composites, including the direct integration of MOF particles onto existing textiles^[^ ^21^, ^26^, ^37^ ^]^ and the formation of MOF/textile composites from MOF/polymer (or precursor) mixtures.^[^ ^3^, ^36^, ^38^ ^]^ Both methods present unique advantages and drawbacks. For instance, integration on preexisting textile fibers usually does not require complicated experimental setups but requires compatibility between MOF particles and textiles, limiting the possibility of suitable combinations. On the other hand, the formation of MOF@polymer composites in a one‐pot synthesis method can expand the range of possible MOFs and polymers, but the formation of these polymer fibers usually requires advanced fabrication tools, which limits their development. In the following section, we will discuss recently developed methods (**Table** **1**) to prepare MOF/textile composites and discuss how researchers address challenges encountered during the fabrication process.

**Table 1:** Summary of methods for preparing MOF/textile composites and their corresponding CWA and simulant detoxification performances under different reaction conditions. This table is divided into three parts (blue, red, and purple lines) based on functionality.

Samples	Fabrication Methods	Mass Loading [wt%]	CWAs/Simulants	Reaction condition	Base Buffer	*t*_1/2_ or conversion	Refs.
*MOF‐based composites for nerve agents and simulants*							
MOF‐808/PAN^a)^/PDA fiber membrane	Electrospinning and solvothermal MOF growth	8	DMNP	Aqueous solution	0.15 m NEM^b)^	≈1 min	[58]
UIO‐66//HEA^c)^ composites	3D photoprinting	10	DENP	Aqueous solution	2.2 wt% NEM	N/A	[47]
MOF‐808/SiO_2_ nanofiber aerogels	Sol–gel electrospinning and freeze drying	5	DMMP	Aqueous solution	0.15 m NEM	5.29 min	[39]
UIO‐66/P(MEMA)_3_/cotton	Drop casting	N/A	DMNP	65% RH,^d)^ Solid state	0.45 m morpholine group	80% (24 h)	[49a]
NU‐1000/PDMAM^e)^/PP^f)^	Free radical polymerization and drop casting	N/A	DIFP	Aqueous solution	–	66% (24 h)	[41a]
MOF‐808/PDMAM/PP						≈97% (24 h)	
UIO‐66‐NH_2_/PET^g)^ fabrics	roll‐to‐roll vapor synthesis	≈14	GD	50% RH, Solid state	–	100% (24 h)	[59]
			DMNP	Aqueous solution	0.45 m NEM	≈18 min	
UIO‐66‐NH_2_/PET fabrics	Sorption‐vapor synthesis	13	DMNP	Aqueous solution	0.15 m NEM	≈13 min	[35b]
			GD	50% RH, Solid state	–	2.5 h	
MOF‐808/cBPEI^h)^/PET fabrics	Dip coating	40	DMNP	50% RH, Solid state	— —	60% (1 h)	[35a]
			GD			90% (24 h)	
MOF‐808 IO film	Hard templated synthesis	N/A	DMNP	Aqueous solution	0.4 m NEM	0.84 min	[51]
UIO‐66‐NCS/RGO^i)^ aerogel	In situ growth and freeze‐drying	55	DMNP	Aqueous solution and simulated solar light	0.45 m NEM	1.3 min	[26e]
Imidazole@defective hierarchical porous UIO‐66/PET fabrics	Ligand‐selective pyrolysis and drop casting	13.2	DMNP	Pure water	–	1.8 min	[49b]
				55% RH, Solid state	–	19.6	
UIO‐66‐NH_2_‐PS/cotton‐COOH composite	Hot press	7.9 (based on Zr metal)	DMNP	Aqueous solution	0.45 m NEM	≈59.6 min	[52]
UIO‐66/PMMA‐BPEI^k)^/cotton	Solution casting	40	DMNP	Aqueous solution	–	0.14 h	[49a]
				99% RH. Solid state	–	4.8 h	
			GD	60% RH, Solid state	–	100% (4 h)	
UIO‐66‐NH_2_/PP	Sorption‐vapor synthesis	5	DMNP	Aqueous solution	0.45 m NEM	≈29 min	[61]
MOF‐808/CNF‐PQ^l)^	Membrane casting, self‐assembly and in situ growth	60	DMNP	Aqueous solution	0.45 m NEM	≈38 min	[38d]
UIO‐66‐NH_2_/PAN nanofiber	Colloidal syringeless electrospinning	50	DIFP	Aqueous solution	–	60% (2 h)	[46b]
UIO‐66‐NH_2_/TiO_2_/PP	Atomic layer deposition and solvothermal MOF growth	≈14	DMNP	Aqueous solution	NEM	4.9 min	[35b]
			GD	50% RH, Solid state	–	177 min	
OA^m)^‐UIO‐66‐NH_2_/PAN	Electrospinning	71	VX	80% RH, solid state	–	≈93.2% (3 h)	[26a]
MOF‐808/BC^n)^ composite sponge	Biosynthesis	60	DMNP	Aqueous solution	0.4 m NEM	<1 min	[21]
UIO‐66‐NH_2_/cotton	Microwave‐assisted synthesis	7.4	DMNP	Aqueous solution	0.45 m NEM	≈30 min	[62]
UIO‐66‐NH_2_/PA‐6		6.3				≈ 30 min	
UIO‐66‐NH_2_/PAN@CNT^o)^ nanofiber	Electrospinning and spraying MOF	85.7	DMNP	Aqueous solution	0.45 m NEM	0.85 min	[48]
UIO‐66/TOCN^P)^ aerogel	Crosslinking and freeze‐drying	54	DMNP	Aqueous solution	0.45 m NEM	0.7 min	[63]
UIO‐66‐NH_2_/PUU	Electrospinning	50	DMNP	Aqueous solution	0.45 m NEM	3 min	[38j]
UIO‐66/AC fabrics	Layer‐by‐layer MOF growth	0.16	DIFP	Aqueous solution	–	80% (24 h)	[37e]
UIO‐66//Nylon 6 nanofiber	Electrospinning and electrospray	56	DMNP	Aqueous solution	0.45 m NEM	0.92 min	[64]
*MOF‐based composites for blister agent simulant (CEES)*							
MOF‐808/PS‐PU fiber sponge	Solution blow spinning and solvothermal MOF growth	N/A	CEES	Acetonitrile solution	–	97.7% (72 h)	[32]
MOF‐808/BPI^j)^ nanofiber	Electrospinning and solvothermal MOF growth	N/A	CEES	≈0% RH, Solid state	–	90.9% (24 h)	[36a]
UIO‐66‐NH_2_/PAN	Electrospinning and solvothermal MOF growth	32.62	CEES	≈0% RH, Solid state	–	60.66% (0.5 h)	[65]
*Bifunctional MOF‐based composites*							
UIO‐66‐NH_2_/MIL‐101(Cr)/PAN fabrics	Electrospinning and soak‐dipping	N/A	DMNP	Aqueous solution	0.45 m NEM	2.8 min	[66]
			CEES	Ambient air	–	90% (4 h)	
MOF‐808/Zr(OH)_4_/PAN nanofiber membrane	Electrospinning, hydrolysis and solvothermal MOF growth	20.6	DMNP	Aqueous solution	0.45 m NEM	1.19 min	[43]
			CEES	50% RH, Solid state	–	89.3% (12 h)	
PCN‐222/PP textile	Template‐free growth	N/A	DMNP	Aqueous solution/blue light	0.45 m NEM	6 min	[13d]
			CEES	Soluble O_2_ in methanol with blue LEDs	–	27.5 min	

Notes:

^a)^ PAN: polyacrylonitrile;

^b)^ NEM: *N*‐ethylmorpholine;

^c)^ HEA: 2‐hedroxyethyl acrylate;

^d)^ RH: relative humidity;

^e)^ PDMAM: poly(2‐(dimethylamino)ethyl methacrylate);

^f)^ PP: polypropylene;

^g)^ PET: poly(ethylene terephthalate);

^h)^ cBPEI: crosslinked branched polyethyleneimine;

^i)^ RGO: reduced graphene oxide;

^j)^ BPI: polymer by crosslink poly^[^ ^20^ ^]^ and poly(polyacrylic acid);

^k)^ PMMA‐BPEI: poly(methyl methacrylate)‐branched poly(ethyleneimine) copolymer;

^l)^ CNF‐PQ: biofilm‐inspired curli nanofiber with poly(vinylidene fluoride)‐grafted poly(quaternized 4‐vinylbenzyl chloride) polyelectrolyte;

^m)^ OA: oleic acid;

^n)^ BC: bacterial cellulose;

^o)^ CNT: carbon nanotube;

^p)^ TOCN: TEMPO‐oxidized cellulose nanofiber; — stands for nothing involved; N/A stands for not available.

**Figure 3:**
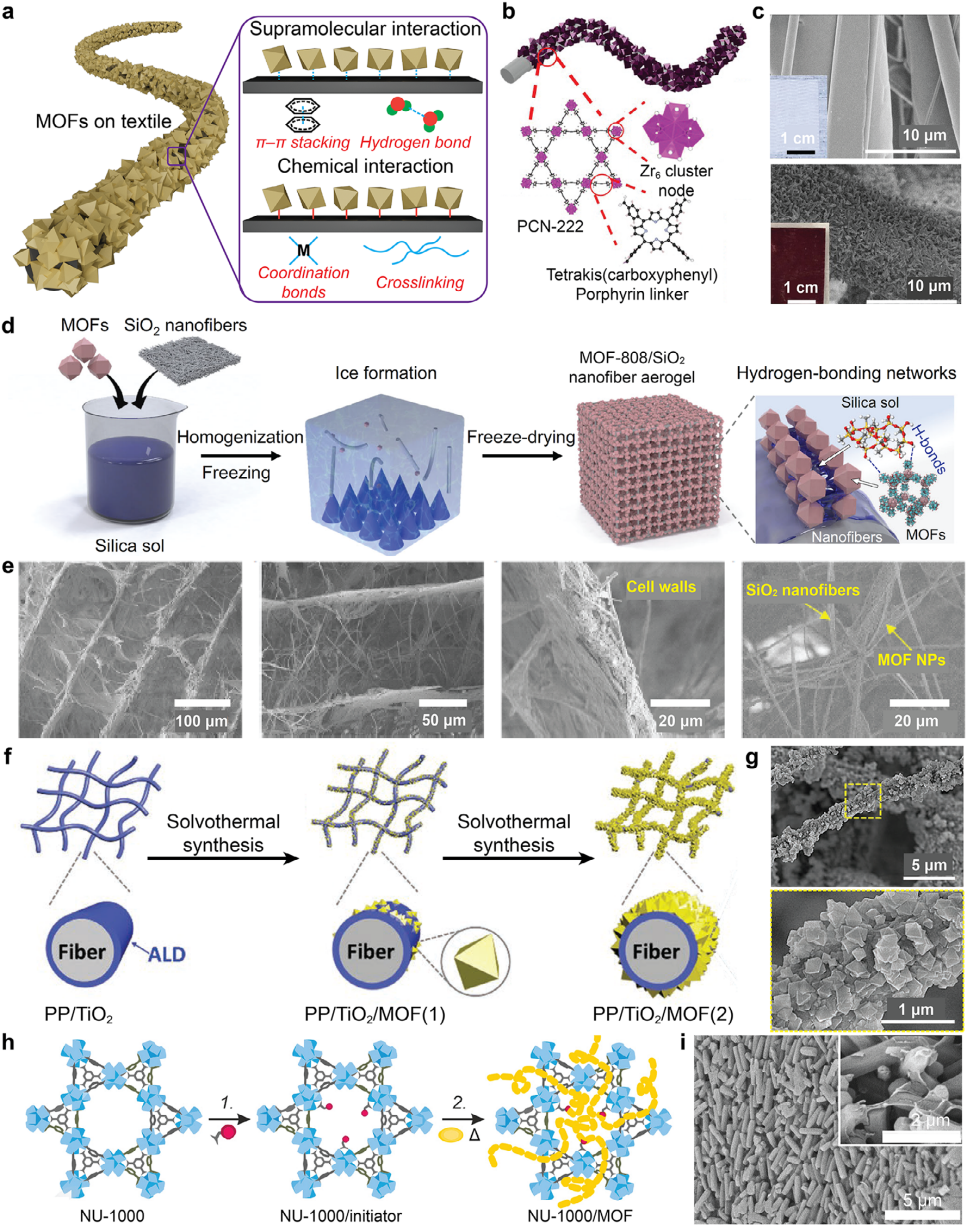
The integration of MOF particles on existing textiles for developing MOF/textile composites. a) The schematic illustration of the integration of MOFs on the surface of a textile with high mass loading and surface coverage using supramolecular and chemical interactions, b) The direct synthesis of PCN‐222 on PP textile via hydrophobic interactions and π–π stacking between the organic linker and PP fiber; c) the SEM images of corresponding PCN‐222/PP textiles; d) the preparation of MOF‐808/SiO_2_ nanofiber aerogel by H‐bonds between MOF‐808 and SiO_2_; e) the corresponding SEM images of MOF‐808/SiO_2_ nanofiber aerogel; f) the synthesis of UiO‐66/TiO_2_/PP textile with the assistance of ALD adhesion layer and solvothermal synthesis; g) the SEM images of UiO‐66/TiO_2_/PP textile after two rounds of solvothermal syntheses; h) the in situ grafting of polymers on MOFs to achieve crosslinking; i) SEM images of the NU‐1000/PMMA/PP textiles. (3b–i) were adopted from published works with some modifications: b,c) Reproduced with permission.^[^ ^13^ ^]^ Copyright 2021, John Wiley and Sons; d,e) Reproduced with permission.^[^ ^39^ ^]^ Copyright 2023, Spring Nature; f,g) Reproduced with permission.^[^ ^35^ ^]^ Copyright 2022, John Wiley and Sons; i) Reproduced with permission.^[^ ^41^ ^]^ Copyright 2023, Royal Society of Chemistry.

The integration of MOF particles on the surface of fabrics and textiles is challenging because of the incompatibility between textile fibers and MOF powders. For example, MOFs are hybrid inorganic–organic materials with high crystallinity and strong coordination bonds that connect inorganic nodes and organic linkers. In contrast, most textiles are composed of amorphous inorganic fibers (e.g., glass wool) or organic polymers (e.g., polystyrene, cellulose, etc.,) with low crystallinity, displaying a large surface property difference between MOF and fabrics. Therefore, creating interactions between MOF crystallites and textiles is necessary and can be achieved either through non‐chemical supramolecular interactions, such as hydrogen bonding^[^ ^39^ ^]^ and π–π stacking interactions,^[^ ^13^ ^]^ or through the formation of coordination bonds^[^ ^40^ ^]^ that result from metal–ligand interactions and crosslinking by polymerization^[^ ^38^, ^41^ ^]^ (**Figure** **3**a). Coordination bonds in these systems form through one of two mechanisms: 1) organic terminal groups on textile surfaces coordinate with metal centers on the MOF surface; or 2) metal centers on textile surfaces coordinate with dangling ligands on MOFs. This distinction requires different considerations during the fabrication process. For instance, Barton et al. reported a direct MOF growth method on a polymer surface by increasing the non‐chemical interaction between the organic linker and polymers^[^ ^13^ ^]^ (Figure 3b). In their work, the Zr‐MOF PCN‐222, synthesized via coordination of the carboxylic acid linker tetra(4‐carboxyphenylporphyrin) (TCPP) with a Zr_6_ node, directly grows on the surface of polypropylene (PP). This was achieved with uniform surface coverage, as shown in scanning electron microscopy (SEM) images (Figure 3c), and tunable crystal sizes that range from 50 to 500 nm. The enhanced supramolecular interactions between the relatively more hydrophobic TCPP units and hydrophobic PP play a key role in hybridizing these two materials to achieve dual functionality for detoxification of nerve agents and blister agents simultaneously.

Similarly, Yan et al. reported that by introducing trimethoxymethylslane‐derived silica (SiO_2_) sol on silica nanofibers, the hydroxyl terminal groups on the silica sol provide hydrogen bonding (H‐bond) sites for MOF‐808 particles with a Gibbs free energy change of −23 kJ mol^−1^, achieving interphase cross‐linking networks with superelastic hierarchical aerogels shaped by freeze drying methods^[^ ^39^ ^]^ (Figure 3d,e). With favorable interfacial interactions between MOF‐808 particles and silica aerogels, the optimized composites exhibit efficient adsorption and decomposition performance against CWAs either in liquid or aerosol forms and exhibit a *t*_1/2_ of 5.29 min toward hydrolysis of the organophosphorus nerve agent simulant dimethylmethylphosphonate (DMMP). This composite also achieved a dynamic breakthrough of 400 L g^−1^, which can be ascribed to the preserved MOF structure, van der Waals barrier channels, minimized diffusion resistance (≈41% reduction), and stability over a thousand compressions.

However, researchers employing this binding‐free, supramolecular chemistry‐based design strategy must consider the chemical structures of MOFs and the corresponding fabric substrates to achieve enough adhesion between textile and MOF powders. Since only a limited number of combinations can be achieved, there is no universal method to integrate MOF on fabrics. Additionally, the constituents for most wearable textiles are typically chemically inert and structurally rigid to maintain durability and prevent contamination from ambient environments. For example, the chemically inert benzyl rings on the backbones of polystyrene fibers display limited interactions with MOF particles. Therefore, incompatibilities between fabrics and MOFs presents a major hurdle for the development of robust MOF/textile composites. To address this issue, surface functionalization of fabrics, which is a strategy used in many studies elsewhere,^[^ ^42^ ^]^ can potentially increase the fabric affinity toward guest materials (i.e., MOFs). In terms of MOF‐based fabrics, the introduction of metal oxides as adhesion layers on fabrics via atomic layer deposition (ALD)^[^ ^40^ ^]^ or wet‐chemistry overgrowth^[^ ^43^ ^]^ can enhance the connection between fabrics and MOF powders. This is achieved by introducing metal–linker interactions that act as nucleation sites for subsequent MOF growth during solvothermal treatment, as demonstrated by the formation of UiO‐66‐NH_2_/TiO_2_/PP composites^[^ ^35^ ^]^ (Figure 3f,g). Therefore, by modifying the surface properties of fabrics or textiles, both the surface coverage and mass loading of MOFs are significantly increased to afford MOF/fiber composites with enhanced catalytic activity, as discussed extensively in the previous reviews.^[^ ^3^, ^4^, ^44^ ^]^

As an alternative approach, MOFs can be integrated onto fabric surfaces by cross‐linking using post‐polymerization methods. Recently, Pander et al. reported the in situ introduction of initiator to the Zr_6_ clusters of NU‐1000 nodes and polymerized polymethyl methacrylate (PMMA), achieving the uniform coating of MOF on PP textiles^[^ ^41^ ^]^ (Figure 3h,i). The chemical bonds between MOF ligands and the polymers increased the connectivity and the strength of MOF/textile composites, serving as an efficient bonding agent to increase the mass loading in comparison to those unmodified counterparts. All the above methods leverage chemical interactions between the MOF and the fabric to lock the MOF in place, expanding the library of possible MOF/fabric combinations. Apart from this advantage, however, since active MOFs are only dispersed on the surface of fabrics where an adhesion layer exists, it may approach the upper limit of an effective mass loading, or enable MOF crystal aggregation, which would inhibit the permeability toward water and air. As shown in our reported work, Ma et al. demonstrated that during the preparation of MOF‐808 particles on textiles with different mass loadings, a *t*_1/2_ for DMNP hydrolysis of <0.5 min is achieved with the mass loading of 6.5 wt% MOF‐808, which is comparable to that obtained when using powdered MOF‐808 under otherwise analogous conditions; however, when 22 wt% mass loading of MOF‐808/textile composites were used, the *t*_1/2_ increased to 3 min. This counterintuitive observation may be ascribed to the greater accessibility of DMNP to the Zr_6_ clusters in the composite with the lower loading as it displays a thinner MOF coating layer.^[^ ^45^ ^]^ A similar phenomenon has also been observed in the biosynthesis of MOF‐808/bacteria cellulose (BC) sponge systems.^[^ ^21^ ^]^ The hydrolytic activity toward DMNP increases with decreased MOF loadings, which can be attributed to enhanced diffusion of the agent through the catalyst by higher external surface area‐to‐volume ratios that occur at lower mass loadings (i.e., a larger ratio of active sites are located on the external surface of the particle compared to that of a particle with a higher MOF loading). Furthermore, even with same metal oxide adhesion layer, the affinity between the metal oxide and the textile differs,^[^ ^40^ ^]^ resulting in different surface coverages and mass loadings, as suggested by PP@TiO_2_ fibers with different hydrophilic properties. Therefore, to determine the best mass loading of specific MOFs on a specific fiber, systematic investigations must be conducted, which may include enhanced screening methods like computational simulations (i.e., artificial intelligence (AI) or machine learning, (ML)) or high‐throughput synthesis methods (i.e., automated robotic systems).

**Figure 4:**
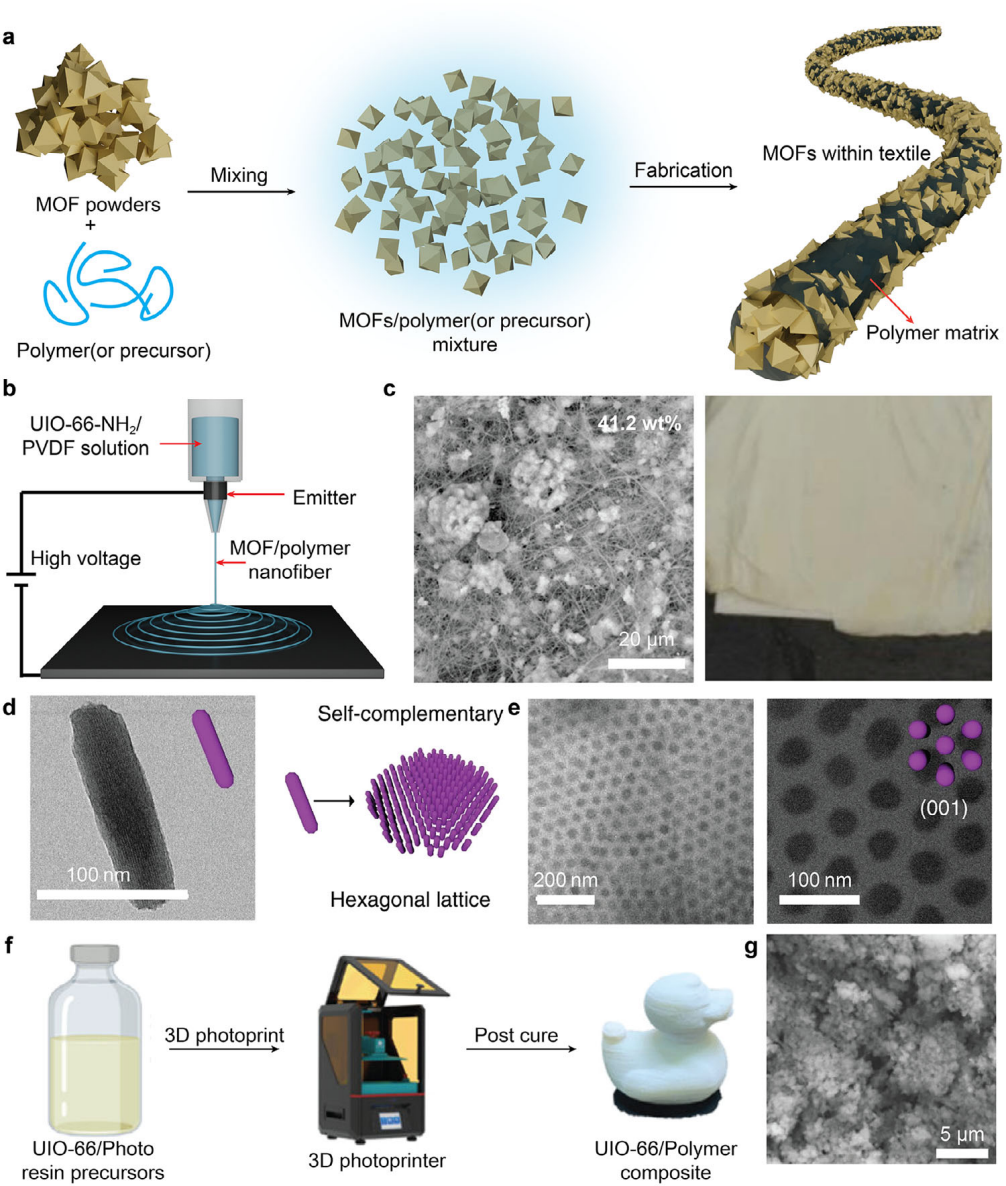
Direct fabrication of MOF/textile composites from MOF particles and polymer (or precursor) mixture. a) Schematic of direct fabrication of MOF/polymer textile by mixing MOF powders and polymer or its precursor followed by various fabrication methods; b) illustration of electrospinning; c) SEM and optical images of the electrospun UiO‐66‐NH_2_/PVDF textile with MOF mass loading of 41.2 wt%; d) TEM image of PCN‐222 used for self‐assembly of e) 2D hexagonal superlattices with long range order by base pair matching of complementary DNA chains; f) preparation of UiO‐66/polymer composite by 3D photoprint starting with UiO‐66/photo resin precursors and post curing process; g) UiO‐66 particles migrate to the polymer surface because of the phase separation behaviors during curing. (4c–g) Were adopted from reported work with some modifications: c) Reproduced with permission.^[^ ^38^ ^]^ Copyright 2017, American Chemical Society; d,e) Reproduced with permission.^[^ ^50^ ^]^ Copyright 2020, Spring Nature; f,g) Reproduced with permission.^[^ ^47^ ^]^ Copyright 2024, American Chemical Society.

On the other hand, instead of undergoing tedious post‐modification and engineering procedures, the direct fabrication of MOF‐based fabrics through electrospinning,^[^ ^26^, ^38^, ^46^ ^]^ 3D printing,^[^ ^47^ ^]^ and spray/dip‐coating^[^ ^35^, ^38^, ^48^ ^]^ or dropcasting^[^ ^41^, ^49^ ^]^ of MOF/polymer composites presents another viable method. By mixing pre‐synthesized MOF particles with the polymer (or precursor) matrix and introducing an external stimulus (e.g., high voltage, light, or mechanical forces), MOF/polymer textiles can be synthesized directly with high quality and efficiency (**Figure** **4**a). Through this route, the mass loadings, types of MOFs, and the polymer matrix can all be tuned based on the desired application, rendering this a more customizable method for designing functional textiles. For example, the Peterson group used an electrospinning method to integrate UiO‐66‐NH_2_ with a polyvinylidene fluoride (PVDF) matrix, achieving a MOF mass loading up to 41.2 wt% (Figure 4b,c).^[^ ^38^ ^]^ This “MOFabric” composite exhibits a low *t*_1/2_ of 131 min toward GD hydrolysis relative to that of the pristine powdered MOF (*t*_1/2_ = 315 min). The enhanced performance can be attributed to the use of PVDF, which promotes the dispersion of GD through the fiber. It is worth noting that for this direct fabrication method, varying the ratio of MOF and polymer affords control over the catalytic and mechanical properties of the composite. In general, lower polymer loadings yield fabric‐like composites with poor durability and integrity, while an excess of polymer matrix may result in blockage of the pores within the MOF particle through infiltration, decreasing the catalytic efficiency.

In addition to the necessity of tuning MOF loading within textiles, another important consideration is the dispersity of the MOF particles on the textiles both on the surface and within the fiber, which affects uniformity of their catalytic and mechanical properties. Homogeneous distribution of MOF particles is highly desirable. To achieve composites with uniform dispersity, precise grafting of surface agents on MOF particles presents a promising strategy. This approach enables uniform surface chemistry and the formation of well‐aligned superstructures, enabling the effective utilization of the collective properties of MOF particles. Within the self‐assembly community, the Mirkin group reported the use of DNA base pairing on adjacent crystals to mediate the spontaneous arrangement of supramolecular structures. 2D hexagonal superlattices comprised of rod‐like PCN‐222 particles^[^ ^50^ ^]^ (Figure 4d,e) exhibit improved photocatalytic oxidation performance toward CEES degradation in comparison to the bulk PCN‐222 powder. This observation could be attributed to faster diffusion kinetics by way of smaller MOF particle sizes and ordered channels within the superlattices. There appears a higher photosensitization efficiency associated with the 2D superlattice, which supports a greater light absorption and penetration depth than in 3D crystals. Similar behaviors have been observed with the development of inverse opal (IO) MOF films developed by Wang et al.^[^ ^51^ ^]^ In one study, a MOF‐808 IO film with an ordered pore size of 2 µm achieved an impressive *t*_1/2_ of 0.84 min for the hydrolysis of DMNP in solution. This success was attributed to the favorable diffusion of organophosphorus aggregates and the matched dynamic size of DMNP emulsions in water (1–2 µm).

Compatibility between the MOF and target polymer, which can impact the uniformity of the distribution and its reproducibility, cannot be overlooked. However, this compatibility is difficult to achieve in most cases, so inhomogeneous distribution and aggregation of MOFs within a polymer matrix are often inevitable. In addition, maximizing accessible surface area is of paramount importance for heterogeneous catalysts. Even with high MOF mass loadings, the number of active sites and subsequent activity is reduced at the interface between the MOF and polymer. This can be addressed by taking advantage of phase separation while mixing the two materials. As shown by Perera et al., UiO‐66 particles were embedded with high surface coverage on a polymer matrix via 3D photoprinting of powder and photo resin polymer precursors under irradiation with 405 nm light^[^ ^47^ ^]^ (Figure 4f). Due to the distinct properties between MOF powder and polymer, phase separation occurs during the polymer curing process. UiO‐66 catalysts migrate to the surface of the printed materials (Figure 4g), resulting in a 10 wt% UiO‐66@polymer composite that displays comparable catalytic activity toward diethyl‐4‐nitrophenyl phosphate (DENP) hydrolysis compared to that of the UiO‐66 powder alone.

Apart from these well‐developed methods, several new strategies have recently been developed to generate MOF/textile composites that circumvent some of the disadvantages discussed previously. As developed by Turetsky et al., the direct synthesis of UiO‐66‐NH_2_ on functionalized cotton textiles (e.g., cellulose fiber with dangling carboxylic acid groups) can be achieved by a hot pressing^[^ ^52^ ^]^ method with PEG‐based polymers at 200 °C for 20 min. This method can generate homogeneously dispersed MOF/textile composites with high mass loadings, eliminating the need for harsh solvents and modulators. This composite achieves a DMNP conversion rate of 68% after 4 h, which exemplifies a potential advantage in scalable synthesis of MOF/textile composites with high efficiency. Similar to the ALD method, a bio‐mimic method to increase the affinity between UiO‐66‐NH_2_ and an electrospun polyamide‐6 (PA‐6) nanofiber membrane was also developed by the introduction of polydopamine (PDA), a naturally occurring biological adhesive mimics developed in the last decade.^[^ ^53^ ^]^ PDAs are densely functionalized with hydroxyl and amine groups that increase the metal chelation capability to promote heterogeneous nucleation, leading to a conformal growth of a dense and uniform thin film coating.^[^ ^54^ ^]^ Notably, PA‐6@PDA@UIO‐66‐NH_2_ exhibits a *t*_1/2_ for DMNP hydrolysis of nearly 0.5 min, which corresponds to a TOF an order of magnitude higher than that of the pure UiO‐66‐NH_2_ powder under irradiation from simulated solar light.

More recently, Wu et al. found that the adhesion layer can further enhance the catalytic performance of the composite toward hydrolyzing CWAs.^[^ ^43^ ^]^ They developed MOF‐808/PAN fiber using Zr(OH)_4_ adhesion layers by pre‐incubating a Zr salt solution in a PAN solution before electrospinning, followed by hydrolysis. The Zr(OH)_4_ adhesion layer not only promoted the binding affinity between MOF‐808 and PAN fibers, but also participated as a catalyst not only capable of efficiently hydrolyzing the nerve agent simulant DMNP, but also detoxifying the blistering agent simulants CEES through a combination of hydrolysis and dehydrohalogenation pathways (Figure 1e). This finding ushers in a new direction for exploration using other adhesion layers to promote the activity of MOF/textile composites. For example, the use of TiO_2_^[^ ^40^, ^55^ ^]^ or g‐C_3_N_4_^[^ ^56^ ^]^ photocatalysts could potentially enable the removal of CWAs with sunlight in ambient conditions. Similarly, incorporating a MOF in reduced graphene oxide‐based composites^[^ ^26^ ^]^ could leverage local heat changes under solar irradiation to further enhance catalytic activity. All these methods, summarized in Table 1, must be exploited for MOF/textile‐based materials to pave the way for new paradigms in the future development of highly efficient composites.

Although many different methods have been developed to achieve uniform MOF coatings on fabrics or textiles, other considerations require attention, particularly mechanical properties. For instance, the stability of MOF/textile composites under the different strains, such as bending or stretching modes, matters since these materials will undergo different levels of structural deformation during manufacturing and utilization of advanced personal protective equipment (PPE) in practical settings. Therefore, in addition to evaluating chemical properties, researchers must also assess mechanical properties^[^ ^57^ ^]^ of composites. For instance, the strain–stress relationships, or tensile strength, indicates the degree of deformation that MOF/fabric composites can undergo before fracturing. The greater the stress–strain toleration and cycling capacity, the more durable and stable the composite becomes. Analysis of previous studies suggests that the adhesion and binding affinity between MOFs and textiles, as well as the interphase configuration, determine the overall durability and stability of these composites. This is illustrated by MOF‐808/SiO_2_ aerogels, which can tolerate more than 3000 compression tests due to the introduction of favorable hydrogen bonding interactions between MOF particles and the hydroxyl functional groups on SiO_2_.^[^ ^39^ ^]^ However, in most studies, the mechanical properties of MOF/textile composites do not correlate with their catalytic performance toward CWA detoxification, limiting the significance of these well‐developed fabrics. Therefore, it is essential to simultaneously evaluate both the mechanical properties and catalytic activities of newly developed MOF/fiber composites, which may potentially provide deeper insights into addressing potential manufacturing challenges of these materials.

### 1.3 Implementation of MOF/Textile Composites in Practical CWAs Detoxification

In addition to developing MOF/textile composites, it is essential to implement these materials in real‐world protective gear and evaluate their performance. Previous studies have demonstrated that there is little difference between the catalytic activities toward real nerve agents and their simulants, as depicted by MOF/fiber composites in solution.^[^ ^1^, ^32^, ^35^ ^]^ However, it is important to mention that the reactions in both cases proceed with the help of a base, which can present challenges for the practical use of these materials in protective gear. Similarly, the use of UV light and oxidizing agents in the detoxification of blistering agents in solution is also impractical since they will cause secondary damage to humans if used in protective gear. Therefore, maintaining the catalytic activities without using harmful chemicals and light sources is a crucial consideration before implementing these materials for practical applications. Furthermore, in real‐world implementations, other key issues include the lack of sufficient water in gas/solid interfaces required for the hydrolysis of nerve agents and catalyst regeneration, as well as the lack of available oxidation species for blistering agent oxidation. In this context, integrating multiple MOF‐based technologies, including textiles, beads, and wipes, can provide a comprehensive approach to address these challenges in practical scenarios. Improving the capacity for capturing humidity in ambient conditions to facilitate nerve agent hydrolysis and desorption of products, as well as local production of oxidation species to achieve efficient oxidation of blistering agents, becomes fundamentally important. In this section, we will discuss the recent advances in addressing these issues and provide insights into the implementation of MOF/textile composites for practical use.

**Figure 5:**
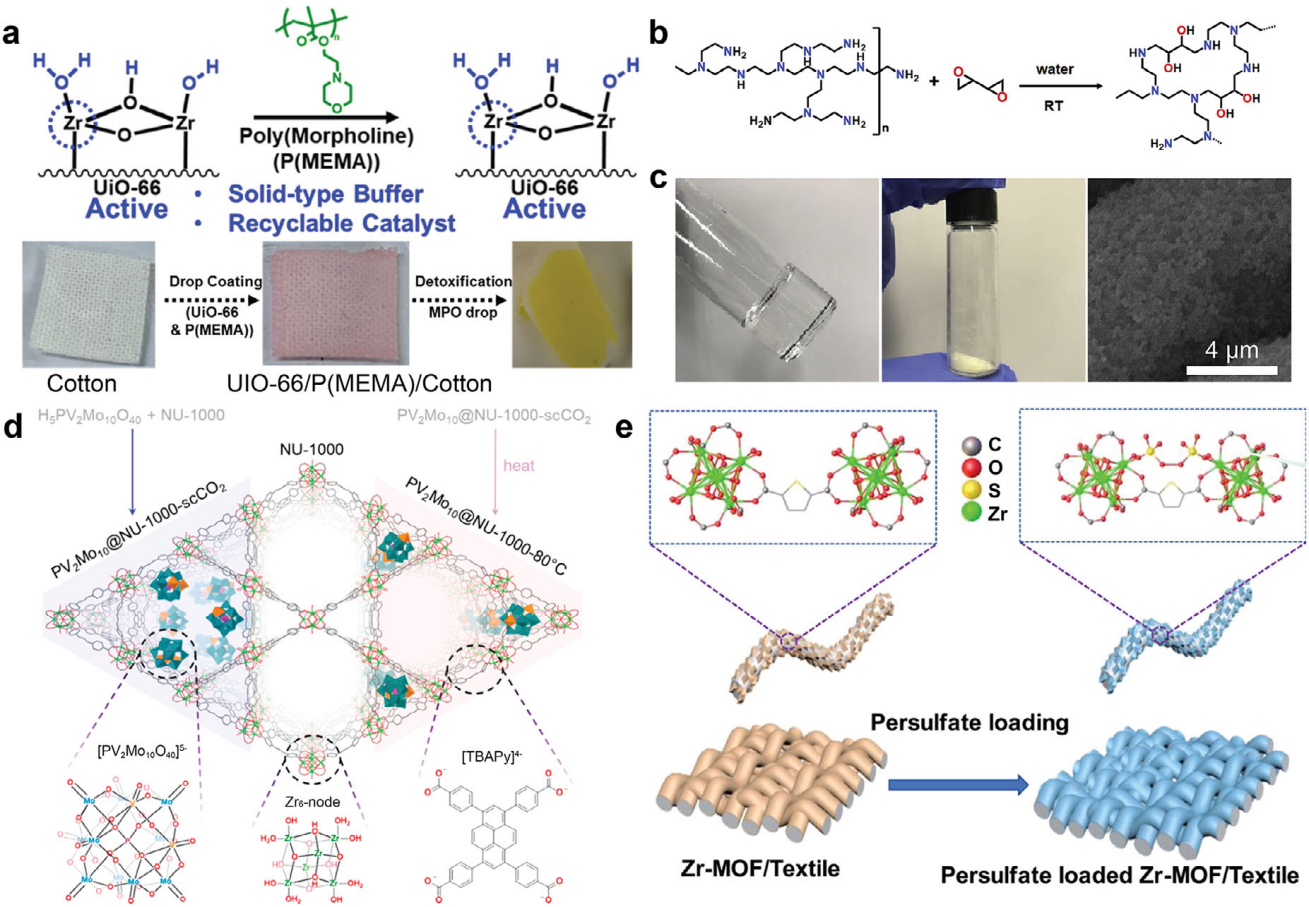
The implementation of MOF/textile composites with consideration of real‐world conditions. a) The use of P(MEMA) polymer in UiO‐66/P(MEMA)/cotton textile acts as a solid buffer and binding agent to integrate MOFs on textile; b) The synthesis of BPEIH; c) photograph of synthesized BPEIH (left), MOF‐808/BPEIH composites (middle) and the SEM images of MOF‐808/BPEIH/PET composites after dip‐coating (right); d) the impregnation of H_5_PV_2_Mo_10_O_40_ POMs within NU‐1000 by different activation methods, results in different local environments within NU‐1000; e) the grafting persulfate ion near the node of MOF‐808; which can be used to produce hydrogen peroxide for removing bacteria. (5a–e) were adopted from reported work with some modifications: a) Reproduced with permission.^[^ ^68^ ^]^ Copyright 2023, American Chemical Society; b,c) Reproduced with permission.^[^ ^69^ ^]^ Copyright 2021, Elsevier; d) Reproduced with permission.^[^ ^75^ ^]^ Copyright 2020, American Chemical Society; e) Reproduced with permission.^[^ ^78^ ^]^ Copyright 2023, American Chemical Society.

For organophosphorus nerve agent detoxification reactions, Lewis acid‐catalyzed hydrolysis is the prevalent strategy employed (vide supra). During hydrolysis, a base is required to maintain high catalytic activities of MOF‐based materials by removing CWA products after hydrolysis to regenerate the active site. As shown in Table 1, *N*‐ethylmorpholine (NEM) is commonly used as the base in the detoxification of organophosphorus CWAs. However, since NEM is a volatile liquid, its long‐time stability and safety in MOF/textile composites raises potential concerns.^[^ ^1^ ^]^ Therefore, replacing volatile, low molecular weight chemicals with less volatile, higher molecular weight polymeric bases or amine‐based dendrimers is a viable solution. As shown in our previous work, Ma et al. reported that catalytic DMNP hydrolysis reactions with the Zr‐MOF NU‐901 and either high molecular weight poly(amidoamine) dendrimers or branched/linear polyethylenimine polymers (B/L‐PEI) achieve comparable half‐lives (≈1 min) to those obtained using an aqueous NEM solution under otherwise analogous conditions.^[^ ^67^ ^]^ The selection of the polymeric base depends on the identity of the target MOF.^[^ ^1^ ^]^ For example, the B‐PEI solution has a lower pH than that of L‐PEI and is better suited for Zr‐MOFs with higher node connectivities (i.e., 12‐connected Zr_6_ nodes of UiO‐66) compared to those with relatively lower node connectivities (i.e., 8‐connected Zr_6_ nodes of NU‐1000 and NU‐901). The introduction of this polymeric amine not only facilitates CWAs hydrolysis, but also works as the binding agent between the MOF powders and textiles, eliminating the need for a polymer matrix that can introduce pore blockage and surface contamination. For example, Seo et al. reported that using the polymeric base poly(2‐morpholino ethyl methacrylate) [P(MEMA)_43_] allows defective UiO‐66 to be incorporated into a textile composite without the need for additional binding agents, resulting in improved performance toward DMNP hydrolysis, with a *t*_1/2_ of 7.7 min 90% retention of activity after three cycles^[^ ^68^ ^]^ (**Figure** **5**a).

In addition to textiles, MOF beads^[^ ^19^ ^]^ have been developed as a replacement for carbon beads in joint service lightweight integrated suit technology gear, which is used for chemical and biological protection by military personnel. MOF beads offer significant advantages over traditional carbon beads, including higher reactivity and the ability to detoxify CWAs rather than merely adsorbing them, reducing the risk of off‐gassing and improving overall protection. Furthermore, MOF‐based materials can be incorporated into decontamination wipes for more efficient removal of chemical agents from surfaces and equipment. Unlike traditional textile‐based wipes, MOF‐infused wipes^[^ ^37^ ^]^ could provide both adsorption and catalytic degradation of toxic chemicals, offering enhanced detoxification capabilities in field decontamination operations. These developments expand the scope of MOF‐based solutions beyond textiles, providing adaptable formats for various environmental conditions and use cases.

To address the challenges faced by MOF/textile composites in ambient conditions with low water content, one proposed strategy incorporates a hydrogel that can retain water for longer periods of time in ambient conditions. Hydrogels are composed of a network of polymer chains that can absorb and trap high capacities of water within their polymeric networks. Our group hypothesized that the local humidity near a MOF particle can be increased via hybridization with a hydrogel, so we integrated MOF‐808 into a branched polyethylenimine hydrogel (BPEIH) by directly mixing in water to form a homogeneous solution, followed by dip‐coating on fibers.^[^ ^69^ ^]^ BPEIH has a high amine density and an abundant supply of water, so it functions as a solid base that mediates the micro‐environment surrounding the MOFs. Even in atmospheric conditions with a relative humidity (RH) of 50%, this hybrid material achieves a *t*_1/2_ of 1 min and a nearly complete conversion (99%) of DMNP after 15 min, which is on par with the performance of MOF‐808 powder in basic aqueous solutions (Figure 5b). More importantly, this MOF/textile composite also shows continued catalytic activity even after aging for 3 months in a sealed vial. Interestingly, in addition to the introduction of polymer hydrogels, researchers have discovered that the MOF itself can form a hydrogel^[^ ^26^, ^28^, ^70^ ^]^ with high water uptake that occurs through a kinetic trap with increased growth kinetics. For example, Zhou et al. reported that a MOF‐808 xerogel^[^ ^27^ ^]^ can be directly formed via a bottom‐up method by decreasing the amount of modulator (a key component to tune the growth rate of MOF particles) whereby small MOF‐808 particles can aggregate to form a gel‐like substance. This method not only decreases the size of MOF‐808 particles, thus imparting higher intrinsic catalytic potency, but also creates a solid material that can be processed into different forms. As suggested by their work, the as‐formed MOF‐808 xerogel shows excellent hydrolysis and dehydrohalogenation activities toward degradation of CEES at 40% RH with a *t*_1/2_ of ≈1 h in solid state, roughly seven times higher than the performance in 0% RH. Furthermore, this degradation performance can be increased to *t*_1/2_ = 13.6 min by hydrating MOF‐808 xerogel in a water solution, followed by air drying (at RH of ≈30%), also indicating the important role of water molecules in the detoxification of blistering agents. From these examples, it is evident that maintaining hydrated and basic sites within MOF/textiles plays a key role in increasing the catalytic activities toward CWA detoxification. All these results highlight the potential for the development of real‐world protection gear.

Although there are many successful cases showing that MOF/textile composites can degrade organophosphorus nerve agents and their simulants in near real‐world use, it is also apparent that research into the oxidation of organosulfur blistering agents and their simulants lags behind since most MOFs lack moieties that can generate oxidation species locally. To address this challenge, similar strategies involving hybridizing MOFs with other materials have been explored. For instance, POMs are anionic metal oxide clusters that contain many active sites and rich redox properties, which makes them potentially suitable for the selective oxidation of organosulfur compounds to non‐toxic sulfoxide products.^[^ ^71^ ^]^ The size of POMs (roughly sub‐nanometer to a few nanometers in diameter) makes them ideal guests for encapsulation in MOFs with suitable pore apertures.^[^ ^72^ ^]^ Previously, the POM [PW_12_O_40_]^3−^ encapsulated within a Cu‐based MOF (NENU‐11) was shown to significantly improve the detoxification of CWAs in comparison to HKUST‐1 and MOF‐5 because of the increased hydrolytic activity on POM active sites, as well as the enhanced adsorptive ability.^[^ ^73^ ^]^ Higher catalytic activity compared to that of the MOF alone can be achieved by POM@MOFs since the synergy between MOF and POMs can further provide new catalytic pathways. For instance, H_5_PV_2_Mo_10_O_40_ has been widely implemented to achieve desulfurization of diesel, and it is believed that by hybridizing this type of POM into MOFs can further increase the oxidative properties of MOF‐based composites.^[^ ^74^ ^]^ Apart from reactivity, one important parameter should consider is the structural stability of POMs within MOFs and it has been accepted that matched pore size of MOFs with the size of POMs can increase the stability of POMs. For instance, our group previously demonstrated that activating POM@NU‐1000 under different conditions, such as supercritical CO_2_ (scCO_2_) or thermally, offers control over the migration of the POM into the different pores^[^ ^75^ ^]^ (Figure 5d). By using elevated temperatures, POMs spontaneously move from the mesopores of NU‐1000 into the micropores, which are thought to be more stable since three surrounding pyrenes interact with POMs. More importantly, CEES can be selectively oxidized into CEESO, the benign oxidation product, by POM@NU‐1000 with oxygen from air and in the absence of light. This strategy can be further extended to other POM@MOF systems^[^ ^76^ ^]^ (e.g., MOF‐808, MIL‐101(Cr), MOF‐808(Ce), etc.) to achieve dual functional materials that are capable of detoxifying HD and GD simultaneously with conversion rates of 97.2% (within 2 h) and 90.8% (within 30 min), respectively. It is important to mention that these POM@MOFs are used in powder from rather than as a MOF/textile composite, but they could potentially be fabricated into textiles via the methods listed in Table 1.

Apart from installing relatively large POMs within the pore of MOFs, installation of small‐molecule species^[^ ^3^, ^34^, ^37^, ^77^ ^]^ inside MOFs can impart new properties without impacting surface area. In order to improve the oxidation properties, Ma et al. incorporated persulfate ions by soaking a Zr‐based MOF on poly(ethylene terephthalate) (PET) cloth in sodium persulfate solution, which coordinate to the Zr_6_ clusters and serve as stable active peroxide precursors, enabling these materials to act as regenerable reservoirs for the slow release of hydrogen peroxide during hydrolysis upon contact with humid air^[^ ^78^ ^]^ (Figure 5d). Although this material was not tested for the oxidation of organosulfur CWAs, it successfully oxidized certain bacteria, which may represent an even greater challenge. These examples highlight the versatile roles of MOFs as they not only serve as catalytic centers for CWA detoxification, but also function as platforms for incorporating other functional materials, suggesting their potential in developing highly efficient protective gear.

While many strategies to combat toxic chemicals have been proposed, the complexity of removing threats of various CWAs and the implementation of these materials in different working conditions remains a great challenge. For instance, the selective permeation and the retention time of CWAs in MOF/textile composites remains pressing.^[^ ^28^ ^]^ For protection gear like masks, it is important that MOF/textile composites selectively block the CWAs and detoxify efficiently while maintaining good breathability. Systematic consideration involving balancing the catalytic activities and permeation is necessary. Ma et al. demonstrated a balance between degradation of CWAs and permeability of materials via a systematic study with multiple variables to develop MOF/textile composite for PPEs.^[^ ^35^ ^]^ Using MOF/crosslinked branched polyethyleneimine (cBPEI)/PET composite fibers synthesized through dip‐coating, they tested the roles of MOF particle size, activation method, MOF loading, and crosslinker amount in the polymer base in its performance. Based on their comprehensive study, they found that air‐dried composites have relatively higher activity compared to those dried by scCO_2_. A higher mass loading of MOF ensures low CWA permeation while still maintaining breathability. It was discovered that incorporating MOF particles in a variety of sizes on both the nano and micro scales is important to increase the retention time of CWAs and intrinsic catalytic activities, while crosslinking polymeric buffer has negligible effects on performance of the composites. These results enabled the development of an optimized system comprised of the air‐dried composite with a mixture of µm and nm particle sizes, a 40 wt% MOF loading, and excellent performance for GD hydrolysis with slow permeation.

### 1.4 Outlooks

Despite the advances in removing threats from CWAs with MOF/textile composites discussed in this review, considerable work remains for these materials to provide sufficient protection under practical conditions. Most of the works summarized demonstrate the potential for implementing MOF‐based composites under practical conditions; however, their effectiveness under varying conditions, such as different temperatures, humidity levels, CWA concentrations, and ambient contaminants, is often overlooked, leaving a significant gap for future development. Additionally, due to the complexity of chemical weapons, achieving comparable detoxification efficiency across different catalytic pathways is essential to ensure continuous protection. In real‐world settings, the best approach involves not only the direct removal of chemical weapons, but also detecting specific CWAs and deploying the most effective protective gear. This requires high‐precision, rapid‐response, and highly sensitive detection capabilities, even at low CWA concentrations. Ideally, protective gear should offer multiple functions, including the detection, sensing, capture, and detoxification of various chemical agents. Achieving this goal necessitates the development of multifunctional materials and the systematic testing of different combinations for optimal protection, which is a promising direction in this field. In the next section, we propose key areas that should be explored in future research.

#### 1.4.1 Developing Multifunctional MOFs for CWA Detoxification under Practical Use Conditions

Given the unpredictable nature and diversity of CWA threats in conflict scenarios, it is often unclear where or how these agents will be deployed. Therefore, designing universal protective gear capable of defending against a range of CWAs is the most effective strategy for ensuring the safety of civilians and personnel. Ideally, the best protection gear should have the capacity to detect CWAs, capture them, and detoxify them using the most effective reaction pathway (e.g., hydrolysis for nerve agents and selective oxidation for blistering agents), while exhibiting excellent selective gas permeability. This could be addressed by either combining several different types of MOFs targeted toward each CWA into a single composite^[^ ^79^ ^]^ or by developing new, multi‐functional MOFs such that one MOF is capable of addressing all types of CWA threats.^[^ ^13^, ^43^, ^74^, ^80^ ^]^ The former approach requires careful consideration of the compatibility between multiple materials to achieve synergistic effects but offers significant advantages over impregnated carbon composites that are currently used, while the latter demands systematic design of the active sites. From Table 1, several cases have demonstrated the possibility of accessing dual functionality for the degradation of both organophosphorus and organosulfur CWAs; however, this direction requires more research to develop dual‐functional MOF materials that simultaneously exhibit state‐of‐the‐art performance for both classes of CWAs. In addition to synthesizing composite materials that provide protection through CWA detoxification, researchers have also demonstrated that MOF/fiber composites can also serve as breathable protective barriers that are highly effective at capturing CWAs under humid conditions, highlighting how these materials can provide comprehensive protection even without immediate detoxification.^[^ ^81^ ^]^

Of critical importance for MOF‐polymer composites is their morphology, particularly how morphology impacts performance in different applications. The morphology is influenced by the compatibility between the MOF and polymer, as well as the resulting interface, both of which can drastically affect the material's effectiveness.^[^ ^82^ ^]^ It is essential to predict these interfacial properties before selecting the appropriate polymer for MOF integration. Developing predictive models or tools to assess compatibility between MOFs and polymers will be key to advancing this field. Moreover, the optimal interface may vary depending on the specific application. For instance, in membrane systems, a strong interaction between the MOF and polymer is necessary to prevent voids that would allow contaminants to bypass the MOF crystals.^[^ ^83^ ^]^ In contrast, in granules or beads, macroscopic voids can be beneficial by facilitating transport from the bulk contaminant phase to the MOF crystals,^[^ ^36^ ^]^ improving performance in detoxification processes. A deeper understanding of how to tune the interface based on these application needs is crucial for improving the overall performance of MOF‐polymer composites. An often‐overlooked factor in MOF‐polymer composites is the effect of processing on composite morphology. Processing conditions, such as solvent choice, temperature, and polymerization techniques, can significantly alter the interfacial properties and, in turn, the composite's activity toward CWA detoxification.^[^ ^84^ ^]^ For example, solvent interactions during processing could either enhance or inhibit MOF dispersion within the polymer matrix, impacting the accessibility of active sites. Tailoring these processing parameters will allow for better control over composite morphology and improved performance in real‐world applications.

Beyond designing functional protective materials that can capture and detoxify CWAs, developing materials that can detect and identify CWAs at low concentrations also presents a key challenge. This capability would not only alert civilians to imminent threats, increasing their chances of survival, but also aid in identifying contaminated areas after chemical weapons have been deployed, facilitating decontamination efforts.^[^ ^2^, ^4^, ^33^, ^37^, ^38^, ^85^ ^]^ Integrating sensing capabilities into MOF/textile composites would provide real‐time detection capabilities, significantly enhancing both immediate protection and long‐term safety. One possible strategy to achieve this involves incorporating a colorimetric detection system to indicate the presence of CWAs. For example, researchers have demonstrated that upon binding of nerve agent simulants to Zr_6_ clusters in Zr‐MOFs, as in the case of UiO‐66‐NH_2_ bound with diethylchlorophosphate, a concentration‐dependent fluorescent response occurs that can be leveraged to detect nerve agents.^[^ ^86^ ^]^ However, this colorimetric system requires excitation of the fluorophore, which may not be suitable for practical applications outside of controlled laboratory settings. Instead, de Koning et al. discovered that incorporating the bidentate dye 5,5‐dithiobis(2‐nitrobenzoic acid) (DTNB) onto the Zr_6_ clusters within a MOF‐808/fiber composite affords a textile material that is capable of the selective colorimetric detection of VX.^[^ ^37^, ^85^ ^]^ Upon pre‐wetting and rubbing a VX‐contaminated surface with the composite, the colorless MOF‐808/DTNB turns bright orange after VX hydrolysis due to the concomitant reaction of the dye with the VX degradation product. Since DTNB reacts selectively with the VX degradation product, this composite material could aid in the identification of the agent used, which is critical for rapidly taking appropriate medical countermeasures, and highlights design concepts that would be most effective for synthesizing new MOF‐based composite materials for the selective identification of CWAs.

#### 1.4.2 AI/ML‐Robotic Arm Systems for High Throughput Screening

Previous studies have demonstrated that the fabrication of functional MOF/textile composites typically requires consideration of many factors, ranging from the MOF identity and loading to the type of base or textile used. Furthermore, balancing detoxification efficiency and the selective permeation of CWA molecules, water, and gas, as well as the durability of the textile, also requires attention. Screening every possible combination within this chemical space would require a huge number of experiments, which is impossible to accomplish in a reasonable timeframe. To circumvent tedious screening tests, high‐throughput screening methods are of particular interest, especially with the help of AI and ML.^[^ ^87^ ^]^ Several pioneering studies have predicted MOF structures and properties through these methods, suggesting that implementing similar systems could effectively narrow the scope of screening tests.^[^ ^87^, ^88^ ^]^ Building from previous reports that have leveraged ML‐based methods to identify optimal conditions for MOF growth on surfaces,^[^ ^89^ ^]^ we propose a possible route to achieve high‐throughput screening using an AI/ML‐robotic system. First, AI training and ML can be used to identify several key parameters for synthesizing MOF/textile composites based on previous studies,^[^ ^90^ ^]^ including the search for catalytic active sites of new MOFs^[^ ^80^, ^91^ ^]^ or other materials like POMs^[^ ^71^ ^]^ and nanoparticles.^[^ ^85^, ^92^ ^]^ Then, the AI/ML can examine the possible combinations of MOFs with polymeric bases and textiles to narrow the screening. Next, the high‐throughput robotic system will screen these conditions to synthesize MOF/fiber composites and run automated tests by mixing pre‐made MOF powder suspensions, polymer solutions, and textiles. Since some experimental parameters may be limited based on the type of robotic system use, we hypothesize that the dip‐coating method will be the most accessible because it requires relatively simple operation without sacrificing material performance. Upon fabrication, the catalytic activities of MOF/textile composites can be measured with in situ NMR or UV–vis spectroscopy to determine *t*_1/2_ of CWA detoxification. This information can be enhanced by determining the complete thermodynamic profiles (*K*_a_, Δ*H*, Δ*S*, Δ*G*) between the MOF and CWA molecules through isothermal titration calorimetry (ITC) measurements, which will provide quantitative insights into the CWA capture performance of these composites.^[^ ^93^ ^]^ Notably, our previous ITC studies into the thermodynamics of CWA binding have illustrated that small differences in thermodynamics can afford stark differences in material properties, such as the efficiency of organophosphorus hydrolysis. After obtaining a relationship between the composites and performance, these results can be further used to train AI or ML models to predict the next round of screening. We believe that iterative training and measurement cycles will narrow down the scope to identify materials with optimal performance, eventually leading to a formula that can guide the development of next‐generation MOF/textile composites for use as protective materials from CWA threats.

## 2 Conclusion

In this review, we discussed recent advances in the development of functional MOF‐based composite materials for the capture and detoxification of CWAs and their simulants, with an emphasis on their practical use in the field. By summarizing recent developments and potential challenges in incorporating MOF particles on textiles, new insights and design rules have been extensively discussed. For protective gear, the utilization of non‐volatile polymeric bases instead of the volatile NEM is of utmost importance to maintain the catalytic activities and stability of MOF/textile composites without introducing harmful chemicals during organophosphorus CWA hydrolysis. On the other hand, the introduction of oxidant precursors is key to promoting the detoxification of organosulfur HD‐based CWAs through the selective oxidation to non‐toxic sulfoxide products. In addition, the catalytic activities of MOF/textile composites also rely on water from ambient air, which can be captured by using a hydrogel with high hydrophilicity or by tuning the reaction pathways by encapsulating POMs or other materials. Furthermore, we emphasize systematic design rules in the implementation of MOF/textile composites by balancing the catalytic activities and permeation of CWAs, highlighting the exploration of different roles involving in the fabrication of MOF/textile composites. We believe that this review offers valuable insights into the integration of MOFs into textiles and serves as a foundation for future development of highly efficient materials, particularly as high‐throughput screening strategies are employed to discover multi‐functional MOF/textile composites.

## Conflict of Interest

The authors declare no conflict of interest.
